# Illinois Opportunity

**Published:** 1898-06

**Authors:** 


### ILLINOIS' OPPORTUNITY.

THE Prairie State has nearly six hundred veterinarians within her borders. Every college of North America and several foreign schools are represented among her number. These veterinarians are not taxed for their occupation, and a large proportion have remunerative practice. Her State is without adequate legislation in many directions; her cities and towns are ill protected with such legislation as would indicate a proper sanitary police system. The State annually loses $100,000 from bovine tuberculosis; an equal amount from glanders; while actinomycosis and many diseases among her sheep, swine, and poultry add thousands of dollars of loss to her livestock industry. A tax of one dollar annually upon each veterinarian would raise such a fund that these facts and these conditions could be presented so forcibly to her people and her legislators that four years of active work would make that State a centre of wise and broad-gauged veterinary sanitary legislation, and an influence throughout the West that would multiply good legislation in every direction and make Illinois the State of States for every other to fashion its legislation after.

This number of the JOURNAL reaches every one of this entire number, and it speaks to you for your district. Will you lead in the movement for your section? Will you give your assistance to this project?

